# Foreign Correspondence

**Published:** 1883-05

**Authors:** 


### Foreign Correspondence.

LONDON, April 5th, 1883.

DEAR JOURNAL—What was said in my last, respecting the admirable facilities for teaching possessed by St. Thomas Hospital School, may also be said with but little variation of the other London schools; all of them are well equipped with everything necessary to give practical instructions. It is apparent that these schools do not make it a primary object to make money out of the students, but rather, the aim is to impart such a knowledge as will enable their men to pass their examinations with credit to themselves and to their college. We see here in operation the plan which the JOURNAL has so strongly urged upon the profession in America, viz.: The existence of examining boards, wholly unconnected with the colleges, composed of men eminent in the profession, which alone have the power of conferring diplomas or licenses to practice. The teaching colleges have no power to confer a degree, much less a license to practice. All of the students must appear before the examining bodies, which are also styled colleges, as, *e. g.*, the Royal College of Physicians of London. They do not, however, give instruction at all, but their diploma or license is conferred upon those students who successfully pass their examinations, and enables the recipient to practice medicine or surgery in any part of the United Kingdom. There is, however, in the present system a serious defect, in that the power to grant licenses to practice is vested in some nineteen different corporations, and there is a lack of uniformity as to the qualifications required. A man, for instance, may be "plucked" by one of the London examining boards, and forthwith present himself before some other licensing body and successfully pass the examination, which gives him a legal status in any part of the Kingdom. This has given rise to the custom of appending to the degree an abbreviation signifying the licensing body. M. D., Lond., is one of the most difficult to obtain, and is considered to outrank the others; and the man who has it, is said to have "a higher qualification." Some of the examining boards, however, are supposed to lower their requirements below a certain standard, and all require at least the following:

"Every applicant for license must produce evidence that he has been engaged in the study of medicine during a period of at least four years subsequent to his registration as a medical student, which period shall include attendance during not less than four winter sessions, or three winter and two summer sessions, at a recognized medical school. He must produce certificates that he has attended the following courses at a university, or at some medical school recognized by the college: Anatomy, six months; Practical Anatomy, six months; Chemistry, six months; Practical Chemistry, three months; Materia Medica and Pharmacy, three months; Physiology or Institutes of Medicine, three months; Practice of Medicine, six months; Clinical Medicine, six months; Principles and Practice of Surgery, six months; Clinical Surgery, three months; Midwifery, three months; Medical Jurisprudence, three months; General Pathology or Pathological Anatomy, three months; Practical Pharmacy, three months. The applicant must also produce evidence of having attended the practice of a public hospital (containing not fewer than eighty beds) during a period of not less than twenty-four months, twelve of which must have been spent in attendance on the medical wards. He must have attended for six months the practice of a public dispensary, or have acted for six months as clinical clerk or dresser in a hospital, or have been engaged for six months as visiting assistant to a registered practitioner. He must produce a certificate of having attended at least six cases of labor under the superintendence of a qualified medical practitioner, and that he has studied vaccination under a competent and recognized teacher. The examinations are conducted partly *viva voce*, partly by written papers.

Candidates for the degree of M. B., from the universities of London, Oxford, Cambridge or Dublin, must have graduated in arts before commencing their medical studies, and all the licensing bodies require that the candidate shall have passed a moderately good preliminary examination—about equal to the Matriculation requirement at Harvard. We would be thankful if we had succeeded in attaining this much, but here there is still a continued and increasing demand for improvements in medical education.

There is now before Parliament a new "Medical Bill," which apparently expresses the sentiments of the progressive men, and receives the hearty support of the medical press. Among other things, it provides for uniformity in requirements, and the establishment of but one licensing body for each of the three Kingdoms; and it is instructive to us to note, that the degree and license-conferring bodies, which would, by the passage of the bill, be shorn of their powers and income, oppose it most heartily, and compact, powerful and well disciplined bodies of this kind are not easily thwarted.

The method of teaching here is quite different from that in American schools, in that didactic lectures occupy an inconspicuous place in the course. One or two a day is considered enough, and it is seriously proposed to do away with them entirely, knowledge of the character imparted by lectures being supposed to be better obtained from the text-books. We, therefore, do not see here the large and convenient amphitheater nor any great body of students collected together at any one time; nor, indeed, do we hear such able and fluent lectures as may be listened to with admiration in America. Talent of that kind is not greatly demanded and is therefore not cultivated.

While one cannot but admire the immense advantages which the student enjoys here, and deplore the lamentable deficiencies of our present system of medical education, still, it is not to be denied that in spite of our educational defects, the profession in America numbers in its ranks our full proportion of men, peers in medical knowledge and ability with those of old England. As to our younger men, so far as my limited observations have gone, I think we have a larger proportion of hard students; men who, recognizing that when their diplomas were obtained they had but commenced the study of medicine, had humility enough to work on, gradually acquiring a wisdom infinitely superior to a mere knowledge of a load of useless formulæ and facts of little value to the active practitioner of the healing art. It has been said that in England knowledge, not wisdom, has become the approved end of education, and cleverness in examinations the "final test of success," which recalls Cowper's differentiation:

> "Knowledge and wisdom, far from being one,
> Have often no connection. Knowledge dwells
> In heads replete with thoughts of other men,
> Wisdom in minds attentive to their own.
> Knowledge is proud that he has learnt so much;
> Wisdom is humble that he knows no more."

There is, however, not the slightest danger of our erring (at least for years to come), in demanding a too technical or severe examination, but there is a reasonable hope that obtaining an independent examining board in each state, we will have found a remedy for the chief of the evils which now disgrace the American profession, and in time a happy absence of that class of ignoramuses with an M. D. to their names which now belittle the American degree.

The March number of the JOURNAL only reached me this week. It was most heartily welcomed, as it was like seeing the face of a familiar friend in a strange land. I have read with much interest Dr. Tremaine's article on "Antiseptic Surgery." By the way, Lister says that Antiseptic Surgery is no longer surgery which excludes the causes of putrifaction, but all those methods now universally employed in some form or other, which more or less *impede* the growth and fermentative action of organisms in the fluids and tissues of wounds. Listerism, is the great principle of *the complete exclusion from wounds of the causes of putrifaction.*

The application of this principle, in the way worked out by him, may be called the Listerian method, and is undoubtedly the best mode now known to accomplish the end desired. That method is best designated by the term "Aseptio," as expressing the result, viz., a complete absence of putrifaction.

It is quite the fashion, at the present time, to assert that the excellent results of the aseptio method depend upon the cleanliness which is supposed to be a concomitant of its use; but in Lister's operations, cleanliness is not a noticeable feature; indeed, he said he was glad to observe accumulation of blood and dirt around the margin of the wound; so long as micro-organisms do not enter this "dirt," it protects the wound from irritation of the antiseptic employed. The spray, he considered the least necessary of all the precautions, because there are fewer organisms in the air than in surrounding objects, and those which fall into the wound may be destroyed by washing the surface with an antiseptic lotion. However, this washing of the wound with an antiseptic lotion is considered objectionable, as being irritant, adding to the danger of carbolic acid poisoning, and not as effective as the spray. In his practice one does not see any irrigation or injection with the solution, but he applies it freely to everything which may come in contract with the wound rather than to the wound itself. This, I think, explains why he does not have any cases of carbolic acid poisoning.

The essentials of his practice have been so well epitomized by Dr. Tremaine, that I will not go into the details of an operation as performed by him. The attention to the minute though vital details, in the carrying out of which it is so easy to slip, is with him, probably, not a matter of thought but habit. If his hand goes out of the spray for an instant, knife and hand invariably go into the stronger solution before again approaching the wound. He now employs the eucalyptal gauze and commends it. As an anæsthetic he uses chloroform, which is administered guttatim on a corner of a towel held an inch or so from the nostrils. He astonished me by saying that he did not believe, as some did, in the danger from chloroform properly administered, and that his direction to his administrator of the anæsthetic was, "Pay no attention to the heart and pulse, watch only the respiration." This rule will do very well with ether, but it is certainly opposed, both by theory and experience, in the use of chloroform.

One cannot but be charmed with Lister's cordiality and readiness to answer questions and to show cases. Nor can he fail to be impressed with the admirable results attained in his wards.

I am inclined to the opinion that the failures which have been reported by eminent men who have employed the Listerian method are due to a non-fulfillment of the necessary details or to attempts to improve upon them. As Volkmann has said, " Many of the unfavorable judgments passed upon this method are due to the fact that surgeons who have not yet learned to experiment with it have made it the subject of their experiments."

I was not a little interested by a visit to the British Lying-in Hospital. The physicians are Heywood Smith and Fancourt Barnes. Since the beginning of 1881, a strictly antiseptic plan of treatment has been adopted. Each patient is delivered under a carbolic spray of one to sixty. She is twice daily syringed out with a two per cent. solution, from the first day after labor. All washing of the genitals is done with a one to sixty carbolic solution In each ward of the hospital there is continually playing a carbolic spray of one to eighty, and the floors are frequently washed out with a carbolic solution. The physicians declare that since the antiseptic precautions have been adopted there is far less feverishness among the patients. Recoveries are more rapid and cases requiring operative assistance recover without a single bad symptom. During the two years there have been but two deaths. One from unavoidable hemorrhage, and one was a widow woman who, when admitted, was extremely weak and emaciated, having been in a starving condition for two months before admission. Three hundred and thirty-two patients were delivered during the two years. Every patient receives three times a day a mixture of quin. sulph. gr. ij, ext. ergot liq. ♏ x, tr. opii ♏ v, acid phosph. dil. ♏ x, aqua ℥i.

Five hundred and thirty-one out-patients were delivered during the past year, with but one death; the same antiseptic precaution being employed.

At the Samaritan Hospital, Knowsley Thornton performs all his operations on the aseptic principle; his excellent results are well known. Perhaps a majority of the surgeons of London employ the Listerian method; but as an impartial observer, I must say that there are not a few eminent and most successful men here who deny the superiority or even the value of Listerism.

Here, however, let me record my own conviction, that however surgeons may differ as to the aseptic method being essential to the best results, or whether it be replaced by a superior method, Lister wears a crown which will grow brighter through the ages, in that his theories and practice here have been the essential cause of that marvelous and happy audacity which, during the past few years, has revolutionized surgery and brought within its sphere of operation all those diseases of joints and of internal organs which were formerly considered beyond the range of practical surgery.

At the Samaritan Hospital, Bantock, who divides the honors with Thornton, has entirely discarded the aseptic method. He told me that he had given it a fair trial, carefully carrying out all the details of the Listerian method, and that his experience with it was unfortunate. While using it he had lost eight cases out of thirty-six ovariotomies. One of the fatal cases had every symptom of acute carbolic acid poisoning. Since discarding the spray, etc., he had performed twenty-three operations and had lost but one case. Keith and Lawson Tait have also, as you know, declared that they get better results without it.

In general surgery, Savory at St. Bartholomew's, and Davy at Westminster, have certainly markedly successful results in their practice, and yet, they do not embrace the aseptic theories or methods. Davy says that Lister obtains his good results not from the use of the Listerian method, but because he is an excellent and careful surgeon and gives his cases much personal attention; but that Listerism has done injury by causing surgeons to belittle, or even ignore the *vis medicatrix naturæ*.

Davy employs the open air treatment of wounds. His results appear to be very good indeed. It is very interesting and instructive to accompany him in his rounds and witness his operations. He has the genius of a "Yankee" for invention, as displayed in numerous surgical mechanisms. He has used his lever for making pressure through the rectum upon the iliac artery in amputations at the hip-joint, now, in forty-two cases. The largest amount of blood lost in any one operation was five ounces, and the least one ounce. The mortality was forty per cent. Its employment is not without danger, as Mr. Davy exhibited one specimen, obtained by a post mortem in a fatal case, which showed that the coats of the rectum had been perforated.

For performing Ogston's operation for genu valgum, and his own very successful method of treatment for bad cases of talipes varus, by the removal of a wedge-shaped portion of the tarsus, he has devised several very ingenious instruments which greatly simplify the operations. He takes plaster casts in all his severe cases of club-feet, before and after operations, and can thus exhibit the result of treatment.

I had intended in this letter to have given you some notes from the "special" hospitals for diseases of the skin, urinary organs, etc., in which I am more particularly interested, but I have, I fear, already made this letter too long and must leave them for another communication.

I had the pleasure of meeting here Dr. A. J. Steele, one of the editors of the *St. Louis Courier of Medicine*, who is known to a great many of your readers, having formerly been Demonstrator of Anatomy in the Buffalo College. All the Americans whom I have met here express themselves as highly pleased with the opportunities for study and observation which London affords, and I must personally express my appreciation of the great kindness and attention which one receives from the great teachers here. They will not only give themselves trouble to show cases and explain methods, but put one under obligations by a hospitality which I fear is not as often shown to the stranger in America.

